# Next of kin’s experiences of involvement during involuntary hospitalisation and coercion

**DOI:** 10.1186/s12910-016-0159-4

**Published:** 2016-11-24

**Authors:** Reidun Førde, Reidun Norvoll, Marit Helene Hem, Reidar Pedersen

**Affiliations:** Centre for Medical Ethics, Institute for Health and Society, University of Oslo, Oslo, Norway

**Keywords:** Ethics in clinical practice, Law, Confidentiality, Qualitative

## Abstract

**Background:**

Norway has extensive and detailed legal requirements and guidelines concerning involvement of next of kin (NOK) during involuntary hospital treatment of seriously mentally ill patients. However, we have little knowledge about what happens in practice. This study explores NOK’s views and experiences of involvement during involuntary hospitalisation in Norway.

**Methods:**

We performed qualitative interviews-focus groups and individual-with 36 adult NOK to adults and adolescents who had been involuntarily admitted once or several times. The semi-structured interview guide included questions on experiences with and views on involvement during serious mental illness and coercion.

**Results:**

Most of the NOK were heavily involved in the patient’s life and illness. Their conceptions of involvement during mental illness and coercion, included many important aspects adding to the traditional focus on substitute decision-making. The overall impression was, with a few exceptions, that the NOK had experienced lack of involvement or had negative experiences as NOK in their encounters with the health services. Not being seen and acknowledged as important caregivers and co sufferers were experienced as offensive and could add to their feelings of guilt. Lack of involvement had as a consequence that vital patient information which the NOK possessed was not shared with the patient’s therapists.

**Conclusions:**

Despite public initiatives to improve the involvement of NOK, the NOK in our study felt neglected, unappreciated and dismissed. The paper discusses possible reasons for the gap between public policies and practice which deserve more attention: 1. A strong and not always correct focus on legal matters. 2. Little emphasis on the role of NOK in professional ethics. 3. The organisation of health services and resource constraints. 4. A conservative culture regarding the role of next of kin in mental health care. Acknowledging these reasons may be helpful to understand deficient involvement of the NOK in voluntary mental health services.

**Electronic supplementary material:**

The online version of this article (doi:10.1186/s12910-016-0159-4) contains supplementary material, which is available to authorized users.

## Background

Next of kin (NOK)^1^ of patients with serious mental illness may have different roles. They often play an important role as informal caregivers contributing to improvement of the patient’s health [1–5]. Furthermore, they are co-sufferers having been deeply involved in their family member’s suffering for years, and may themselves carry symptoms and ailments as a result of what they have been going through [6–8].

Next of kin have often been regarded as a cause of the patient’s mental health problems, [1, 7] or at least part of the patient’s dysfunctional behavior patterns. Accordingly, it may seem rational to create a distance between a suffering patient and the family when the life of the patient becomes so intolerable that they need hospitalisation. Adding to this, the health care professional’s ethics and health law are mainly focused on the patient, informed consent and autonomy, professional care, and confidentiality, while family and social relations are more peripheral or neglected. In Norwegian health law, the role of the next of kin is primarily described as being a representative for the patient when the patient’s mental competence is lacking.

Lack of resources and time limitations may cause suboptimal treatment and care in Norwegian mental health care [9]. One may question whether mental health care, which already struggles with limited resources, should be required to support next of kin in addition to giving necessary treatment and care to a mentally ill person. In addition to resource allocation dilemmas, responsibility for the next of kin’s needs may create other ethical dilemmas for health care personnel related to confidentiality and weighing loyalty to the patient up against the needs of the next of kin [10], e.g. how should health care deal with anxious relatives of patients who do not wish to have their family involved?

A substantial amount of research literature confirms the important but very difficult role of next of kin of seriously ill patients [11, 12]. Better involvement of the patient’s family and network in the treatment may improve the patient’s and the relatives’ health, reduce sick leave rates, and reduce health care costs [4]. Over the last 25 years, several policy initiatives have been taken to increase the involvement of NOK of people with serious mental health problems [13–15]. However, various barriers exist which limit necessary involvement of NOK [7, 8, 16, 17].

When it comes to involvement of NOK during serious mental illness and coercion (e.g. involuntary hospitalisation), there is little knowledge about the NOK’s own perceptions, opinions and experiences of ‘involvement’. This is a problem, since the question of involvement of NOK may be particularly pressing and challenging during coercive treatment. This paper presents NOK of patients exposed to coercive measures’ views on the importance of involvement, their experiences of being involved during hospitalization of their family member and their suggestions for improvement when their family member is involuntarily hospitalised.

## Method

### Study context and design

Norwegian citizens are legally autonomous in health matters at age 16. Before this, parents must consent to treatment, and they are entitled to receive all information on behalf of their child. In cases where the patient is older than 16 and lacks decision-making capacity, the NOK should be asked what they know about the patient’s presumed preferences before decisions are made. To fulfill this representative role, the relatives need to receive all relevant information about the patient’s illness and treatment.

In the Norwegian mental health care legislation, lack of competence is not among the formal criteria for involuntary treatment. The main criterion for using coercive measures is serious mental disorder. Furthermore, one of the two following criteria has to be fulfilled: The psychiatric treatment is necessary due to the potential treatment benefits for the patient (“the treatment criterion”) or the patient is assessed to be a great danger to him/herself (“the risk criterion”) or to others.

Irrespective of the patient’s level of competence, the next of kin have the right to appeal any decision about coercive measures, and must accordingly receive necessary information. This information is limited to what is strictly necessary to complain, i.e. that a formal decision has been made and information about the right to complain, what has been decided, and which conditions in the law that are fulfilled, but no further information, e.g. about the reasons for coercion etc [13]. Thus, the NOK’s right to information in such instances is very limited compared with the information NOK are entitled to when the patient lacks competence to consent.

Formal coercion for adults and adolescents is mainly performed within specialised health services, while community health services can request involuntary hospitalisation. Mental health care in Norway is publicly funded and organised as ‘specialised health services’ – i.e. hospital trusts (hospitals and outpatient clinics) and as ‘community health services’ (e.g. general practitioners, ambulant services, local emergency teams, supported housing).

This study is part of a large-scale project in Norway called ‘Mental health care, ethics and coercion’ (PET), started in 2011. The project aims to explore ethical challenges related to coercion and involvement in care from the perspectives of staff, patients and next of kin using a qualitative design across various settings [18]. Focus group interviews were chosen as the primary method for gathering empirical data because group interactions can stimulate open, democratic discussions about the moral views regarding coercion [19–21].

### The participants

The study consists of three focus group interviews and one individual interview with next of kin to adults and three focus group interviews and one parent interview with parents to adolescents, in total 36 NOK to 33 ill family members. All patients had been involuntarily admitted once or several times. Four adolescents had been hospitalised before the age of 16 on parental consent. The study population was dominated by women. An overview of participants, type of family relations and mental health problems, and types of coercion is shown in Table 1.

**Table 1:** Overview participants

	NOK to adults (*N* = 20)	NOK to adolescents (*N* = 16)
Age of interviewee	30–65 years	35–55 years
Family relations	Father (1) Mother (13) Sister (2) Daughter (3) Female partner to male (1)	Parents (5) Mother (5) Father (1)
Age of sick family members	23–57	16–20
Type of mental health problems	Psychosis/schizophrenia (majority) Bipolar disorder Depression/suicidal thoughts ADHD and Asberger’s syndrom Substance abuse problems	Anorexia nervosa Anxiety/Depression Suicidal thoughts/self harm Psychosis/bipolar episode
Duration of problems	1–20 years	One short episode - 5 years.

### Recruitment and data collection

Participants were interviewed between November of 2012 and March of 2013.

A combination of purposive and convenience sampling was used with broad inclusion criteria. Family members to adults were recruited through local NOK organisations in three counties in south-eastern Norway. Family members to adolescents were recruited from adolescent wards in two hospitals participating in the PET research project.

The focus group interviews, consisting of 4–7 participants, were held in NOK-organisations’ meeting rooms or in the hospital and lasted 3 hours, including breaks. The parent interviews and individual interview were held in private locations. The individual interviews were conducted by one researcher (RN), and the focus group interviews were led by two researchers (RN and RP or MHH) who had different roles, one being the moderator and main interviewer [19–21].

The semi-structured interview guide (see Additional files 1 and 2) included questions on coercion and experiences with and views on involvement during serious mental illness and coercion. Data from the last part of the interview guides are presented in this paper. We used a broad definition of coercion, including formal, informal and perceived coercion and any type of coercive measure (involuntary commitment, physical restraints, involuntary medication etc.). Participants were encouraged to illustrate their views with concrete examples.

### Data analysis

The transcribed interviews were first read to get an impression of the whole, then reread several times paragraph by paragraph, and notes were taken to get a memo of the content (e.g. NOK missing information, feelings of being met with hostility and cooperation with the health care). A special focus was on descriptions of the cooperation between NOK and health care personnel, and the NOK’s suggestions for improvements. Based on the memos, categories were made. Interpretations led to establishment of patterns and links between themes. All authors have read the interviews individually, and have discussed the findings and interpretations.

The results will be presented under four headings; 1) Life as next of kin 2) The importance of involved during serious mental illness and coercion. 3) Experiences with inadequate involvement. 4) Descriptions about useful or good involvement.

## Results

### Life as next of kin

Most of the participants have lived close to the patient for many years, and most of them are deeply involved in the patient’s life and illness. The informal care responsibilities are generally extensive, in particular during aggravation of the illness before an involuntary hospitalisation.

NOK gave vivid pictures of their life with family members with serious mental illness and of their own suffering, anger, anxiety and grief for their family member. They describe feelings of deep loneliness and insufficient social support as they, due to feelings of guilt and shame-or out of loyalty to their loved one-had not permitted themselves to share these negative experiences with their social network. Accordingly, they had a great need to be seen, acknowledged and met by the health care personnel taking care of their family member.

One example of this continuous pressure over years, is the description from the parents of a child with serious eating and self-harm disorders: “*So it is worse than having an infant at home. You have to be on guard almost all the time. You wonder how their mood is today*, *how are things*, *how is the look in her eyes. Yeah*, *you study your child the whole damn time*, *really*! *And you walk around dreading something*, *kind of*…”

Even though there could be conflicts between patients and family members regarding the need for hospitalisation and medication, the participants’ willingness to care for their family member over time, and their strength and creativity in this matter are striking. The person with a serious mental illness might be a burden, but is also a loved one.

### The importance of involvement during serious mental illness and coercion

The NOK had a varied understanding of involvement during involuntary hospitalisation. Their concerns and feelings of responsibility did not cease although health care had formally taken over the treatment responsibility. Being seen, met, included and acknowledged as informal caregivers during hospitalisation is a basic element of involvement. This also included acknowledging NOK’s own needs and interests, e.g. during a crisis related to the involuntary hospitalisation.

As caregivers they wanted to contribute with relevant information about the patient’s illness and needs, and they, in turn, needed general information about the illness, treatment and care, information about the services and routines in the institution. Some NOK also wanted more specific information, however, they generally did not want all the details, recognising that they do not have unlimited rights to be involved in personal matters. Many NOK state that they do not want private information, but need general information which they are legally entitled to, and which may offer relief and make them able to prepare their near future and improve the support they want to offer as NOK: “*I do not want them to breach confidentiality*; *I just need to know what is happening*.” (daughter of adult patient).

NOK described involvement as communication, as a dialogue, rather than a one-way information flow. Involvement and dialogue are particularly important the first time a family member is involuntarily admitted, since this is often very scary. A positive story was told by a mother: “*We felt we were trusted. There was a long meeting*, *with two doctors* (…).. *and my ex and me. And it made such an impression on my ex*, *that he started sobbing*. (…) *it was such a relief to be believed*. (….) *and to be regarded as someone who could give some extra information*, *some background information about our son* …”. (mother of adult patient).

Involvement did not mean treatment responsibility for their mentally ill family member. Many NOK describe that they are given too much responsibility in the treatment of their family member. Most of them state that they need to be just family members.

### Noninvolvement may harm the patient

Preserving or repairing family bonds, during and after aggravation of illness and hospitalisation, was one reason why NOK felt that their involvement was necessary.

Although no longer having the main responsibility is a great relief, they describe how they and their sick family member “lose each other” due to long hospitalisations. This breaking of family bonds is particularly problematic if the family is responsible for practical and emotional support following hospitalisation.

Health care personnel do not acknowledge and request their often in-depth knowledge. NOK describe this lack of interest in their experiences and perspectives as offensive and wrong, and as a threat to the patient: *How much involuntary treatment could have been avoided if they had cooperated with us*? a mother of an adolescent asks. Thus, the professional may miss relevant information which could have revealed that the patient needed intervention at an earlier stage of the illness. When health care rejects this information, and in some instances ridicules them for their points of view, the necessary (extensive) intervention may be given too late.

The NOK also describe how they discover serious side effects of medication before the therapists do, and feel that these observations should be appreciated for the patient’s best interest. When family members identify drug side effects neglected by the therapists, trust is reduced, and may result in the family members taking more (often unwanted) treatment responsibility.

### Experiences with inadequate involvement

The NOK’s narratives of how they were involved and met when their family member was hospitalised due to serious mental illness were related both to voluntary and involuntary treatment. Some NOK tell that their family member wanted no contact during the hospital stay, and that this lack of contact could be very difficult emotionally.

The overall impression in the interviews was that the NOK had experienced lack of involvement or had negative experiences as NOK in their encounters with the health services.

### Offensive and undignified attitudes

Adult next of kin of adult patients gave the most vivid examples of non-involvement. Many describe a system which does not function, a hostile and cold system stripped of humanity and lack of trust. When their family member is admitted against their will, often initiated by them, they become particularly vulnerable. When they are treated like strangers they feel deeply wounded and humiliated. They also describe how frightening the first meeting with the hospital is: “*If someone could have come and taken care of me*. (…). *It was very*, *very frightening the first time we met this insanely scary system. It is immensely frightening in the start*.” (daughter of adult patient).

Instead of being acknowledged, comforted and informed, they often felt rejected and met with hostility, which may increase the family members’ feelings of guilt and anxiety:”* I believe that the biggest problem is the attitudes towards the patients*’ *families* (…) *that they regard you almost as the cause of your son*’*s problems*. (…) *as an additional burden. This is an impossible starting point for communication* … *Because they could also see us as a resource*.” (mother of adult patient).

They describe how health care personnel gave loyalty conflicts between them and the patient as an explanation for not communicating with them, although their needs are modest: “*They find it unethical to cooperate with us*, *unethical to talk to* [*us*]… *who have the responsibility for our daughter*… *It is totally lacking logic and humanity*, *and also lack of respect which I have seen when I visit*, *no one greets us*, *no one offers a glass of water*, *no one asks how are you doing*, *this must be very tough* … *You are not taken seriously*, *you are not met* …” (mother of adult patient).

In all the interviews, dialogue and lack of information are described as major problems. In involuntary hospitalisation, they worry about the consequences that this may have for the patient.

The NOK describe a health care system which is fragmented. Plans are not being made, neither for the hospital stay nor for the time after, or shared with the NOK. According to them, lack of long-term plans also reduces the value and effect of the hospital stay. “*I have experienced that they call on Friday at two o*’*clock*: ‘*Hey*, *your mother will come home today*, *and she cannot be left alone* (…). *Yyou have to look after your mother*.’ *No question* (…) *It is your responsibility*.” (daughter of adult patient).

### Information and the dominating legal focus

The family members describe how they are met with information about legal regulations, in particular the rights to file a complaint about coercive measures on behalf of the patient, and that the therapists put great emphasis on sharing necessary information related to these formulations in the law. This information may feel cold and alienating. They describe health care personnel as “afraid of the law”: “… *what I have received after he was hospitalised is letters*, *actually cold letters saying*: ‘*For your information*, *today S has been physically restrained*.’ *Many letters*, *every time it is like* … (*starts crying*) (…) *They have not talked to me at all about his condition when he is exposed to coercion. Nobody has talked*, *but I get letters and no one to talk to*. (…) *And when I call*, *asking how is he doing*: “*We have to protect his right to confidentiality*.” (…) *Not to know anything*, *not to get through to him*, *to know how he really is doing*. (…) *How can I help improve his situation*, *how can I cooperate*? (…) *How can I help my son*, *be there for him*?” (mother of adult patient).

That 16-year-olds are legally responsible for their health care is seen as wrong by the NOK of young patients who are totally dependent on them and their surroundings. The patient’s dependence on the NOK is often not acknowledged by the health care personnel, who seem to focus on some parts of the law only. Lack of information is especially difficult for these parents who described years of worry and a deep feeling of responsibility for their suffering children who at the age of 16 may be formally responsible for their own health care decisions: “… *A wall came up at the age of 16*. (…) *She is living at home*, *and she is still in school* (…) *and she is my responsibility* … *And I have no say*.” (parent of adolescent).

In all the interviews, the next of kin describe situations in which health personnel seem to justify inhumane action through the law, not using common reasoning, good clinical judgment or humane considerations. Sometimes the health professionals seem to apply the law wrongfully through neglecting clauses in the law, e.g.in the cases in which the patients are not mentally competent, they, according to the participants, wrongfully give confidentiality as an explanation for not involving and informing NOK.

### Experiences with positive involvement

Although some NOK describe health personnel who have tried to take care of them, these stories are rare in the interviews. These exceptions are described as being linked to the personality of the health care worker, to the ability to relate to the patient’s family in a good way, as a sign of proficiency and as a sign of individuals who have managed to keep their humanity intact in an inhuman system: “*Health care personnel are part of the system*, *individuals who do the best they can within the frames they have to work. And we have a fantastic psychologist. He is so positive*, *he lifts people up*.” (mother of adult patient).

The importance of being seen and treated as a human being, with respect is emphasised: “*I remember the first days*, *we visited him every day*, *and I felt it was a place where a lot of people* “*were riding their horses*,” *almost warlike. Heavy things going on in there*! *And then suddenly someone stopped and looked at me*: *Hey*, *there you are*, *how are you*? *And just because someone was caring and asking*, *I started to sob*. (…) *Because they saw me*. (…). *I remember the few times someone looked at me*, *it was heartwarming*, *it meant so much*, *even though it was nothing big*, *just to be seen and smiled at and stuff*”. (daughter of adult patient).

The mother of a 24 year old boy also told that she started to cry when the staff greeted her and asked how she was doing: “*A few times*, *somebody saw me*, *maybe they smiled through the glass door*, *those little things*, *it really warmed me*, *it meant a lot*”. She also talked about having been in dialogues about medication for her son; she was informed, allowed to ask questions, she was involved in discussions about medication and possible side effects. One mother of an adolescent participated in a parent education program where the physician who was responsible said that she could phone him anytime. She felt so relieved about that, but she phoned him only once. However, comments like: “Just call us if you need anything” may be of little help since the system is very often impenetrable, and it may be difficult to find the right person to talk to when you need it. Thus, a contact person in the institution can give stability and facilitate communication. Stability is also strengthened by good communication within the health care team and between different parts of the health care system.

Early establishment of an open dialogue with the patient and the family members about the illness and hospital routines was seen as important for coping, increasing trust, reducing anxiety, and preparing for the time to come after hospitalisation. NOK also described how information must be repeated. The information they get early on, when everything is chaotic, and they are in a state of shock, is easily forgotten.

A mother described that for her it was important to be allowed to see the seclusion room, to be present there and share a slice of bread with her son. It helped the horror images she carried with her.

There was a need for staff to take more active responsibility for the family by calling them, inviting them to meetings, doing home visits or offering help to work more actively with sustaining relationship in more healthy periods. Further, they pointed to the importance of establishing or strengthening a network of family and friends. Networks consisting of health care professionals, family members and friends where they exchange information and take each other’s perspectives were seen as valuable, may improve dialogue and involvement between the parties and strengthen the relationship between patient and NOK. The general practitioner is often a key person in the patient’s network, as is a nurse who is available to the family members.

The mother of an adolescent gratefully tells about how they were helped in their reconciliation work: “*When the time for discharge was approaching*, *my son and his nurse started to map his network*, *and his nurse discovered that he had a hard time in his relationship to me since he felt so bad about how he had behaved towards me. The nurse suggested that the three of us should meet and dialogue about it. She reserved time for it*, *and assisted my son and me in verbalising what was difficult*, *and we reconciled. We would not have coped as well with that on our own*.”

## Discussion

### Weaknesses and strengths in this study

The NOK of adult patients were recruited from NOK organizations which may recruit the most active, but possibly also the most frustrated part of the NOK population. Women dominate this group of participants. The data could possibly be different with a different gender balance. Our material consists of extensive interviews with NOK of a wide variety of patients with serious mental health problems and of different ages. We believe that our study adds knowledge to how NOK of patients who have been submitted to different kinds of involuntary treatment in mental health care want to be involved in the treatment of their family members. This group of NOK often carries a feeling of guilt for subjecting the patient to coercion and therefore their needs are particularly important.

Similar studies outside Norway confirm that NOK to adults as well as adolescents/children also experience lack of, or inconsistent, support and involvement [1, 6, 7, 14], and experience feelings of powerlessness and alienation by the way they are met [22].

### The gap between public policies and practice-possible reasons

Guidelines from 2008 issued by The Norwegian Directorate of Health stress the importance of involvement of NOK in mental health care and they give many practical recommendations on how to do this correctly [13]. The present study indicates that despite many public initiatives and despite evidence of the usefulness of sound family involvement, the NOKs still feel neglected, unappreciated and dismissed.

In the following we will discuss five possible reasons for this in light of our findings. First, the relatives seem to have a broader conception of involvement and emphasize different aspects of involvement than health law does; second, a strong and not always correct focus on legal matters in mental health care; third, very little emphasis on the role of NOK in professional ethics; fourth, organization of health services and resource constraints, and finally a conservative culture regarding the role of next of kin in mental health care. These reasons may also be helpful to understand deficient involvement of the NOK in voluntary mental health services.

1. Different conceptions and priorities
  The interviews reveal that NOK’s conception of involvement covers several “levels”; help to maintain relations and social bonds, being acknowledged as informal caregivers possessing and requiring important knowledge, and needs to being met as vulnerable human beings with their own needs for support, all these requiring communication and dialogue. Health law and health care ethics have mostly focused on the NOKs’ role in decision making, as the seriously ill patient’s representative, or to launch complaints in cases of coercion. In the NOK’s descriptions of involvement this (legal) kind of involvement is barely mentioned.
2. Dialogue based on law
  As mental health care has the rights and protection of the individual patient as its primary aim, knowing how to include the interests of the NOK in a way that does not threaten the individual patient’s legal rights may be difficult. Even when law is interpreted correctly the legal “language” e.g. put forward in letters informing about coercive measures, may be experienced as cold and downgrading. The NOK describe that confidentiality considerations are given as a reason for not talking to and involving the NOK, even when there is a high probability that the patient’s competence to consent is lacking. To this adds that most of the information NOK request is not against law, but general information which may ease NOK’s tension and improve coping. Furthermore, acknowledging the NOK’s role as informal caregivers possessing valuable knowledge, does not threaten confidentiality.
  Norwegian law regulating mental health care may be confusing. NOK have the right to be informed about coercive interventions-but just enough information to be able to make a complaint. If the patient lacks competence to consent the NOK’s right to information is more extensive. However, as lack of competence to consent is not a formal criterion for coercive measures in mental health care, the patient’s competence is rarely assessed and more extensive possibilities to inform the NOK are forfeited. Our data indicate insufficient legal knowledge e.g. about the parent’s right to information for patients between 16 and 18 years where the parents are entitled to information that is necessary to fulfil parental responsibilities (up to the age of 18).
  However, as a main rule, except in cases of coercion, if the patient is competent to consent, Norwegian health law treats the NOK as any stranger. This is a paradox since cooperating health care professionals may share information among themselves without asking the patient. Informal caregivers with heavy responsibilities for the patient’s functioning have no corresponding right to receive information necessary to fulfill their role as informal carers in the patient’s best interests.
3. Ethical considerations
  Professional codes of ethics and health care ethics have, as health law, a strong focus on the individual patient and on decision making. However, as seen in our interviews, NOK of mentally ill patients have needs and roles which go far beyond substitute decision-making. Information and a good dialogue between the caregivers and NOK are vital both for the patient and the NOK. This does not mean that information sharing is an ethically simple issue. There is no straightforward border between relevant information (adapted to each patient’s situation) given in a language which is empathic and understandable for family members and information which is too private or intimidating. One way to solve this, while still respecting patient autonomy, is to discuss with the patient the need to share information with family, when the patient’s illness is not too severe.
  There may be obvious conflicts of interests between patients and their family. Patients may have good reasons to keep a distance to their families. To involve NOK in an ethically acceptable way when NOK have a negative influence on the patient’s mental health may be a delicate balance. Attention and support to these relatives may be a provocation for some patients and must be based on good ethical judgment in each individual case. Neglect or exclusion of the NOK is probably not a good way to deal with family conflicts in cases of severe mental illness. The emphasis on family ethics should be strengthened in the education of health care personnel and in medical ethics [23, 24]. It is thought-provoking that the role of NOK has barely any place in the (extensive) professional ethics rules for Norwegian doctors and psychologists, and a limited place for nurses’ ethics guidelines.
4. The organization of the services and resources
  Fragmentation of mental health care, and lack of cooperation between different levels of health care may hamper the establishment of a network around the patient, which includes the NOK who are willing to be involved. Many of the interviewed NOKs have some positive experiences with family involvement in services that are more focused on voluntariness and cooperation, e.g. community services which in Norway, by and large, have no legal authority to use coercion. Available and feasible ways to improve NOK involvement are not well integrated in many services. If the services are unavailable when the patient is still motivated to get voluntary treatment and care, the NOK may feel forced to become more actively involved than they would like to, including initiating involuntary admission. Lack of available services, being the initiator of coercion, experiences of ineffective coercive measures, and feelings of guilt may create difficult barriers to trust and cooperation between the patient, the NOK, and the professionals.
  Resource limitations may be given as reasons for lack of involvement of NOK. If NOK are met with hostility and a lack of friendly and empathic communication, this may worsen their situation as caregivers. Small changes in attitudes, communication and information sharing between health care personnel and NOK can easily be done within acceptable time limitations. Resource limitations can hardly justify status quo [5]. If health care can help NOK to cope better with the (often heavy) responsibility this may even save resources by preventing hospitalisation. The same goes for active support in necessary repair of family bonds. Emphasis on stability of personnel, on creating good networks within the institution and between primary health care, the family and the institution may, on the contrary, both save resources and protect the interests of the patient as well as the next of kin. Whether resources spent on time consuming, emotional support to NOK individually can be defended, is another question.
5. Changing the culture in mental health care
  Mental health care has been influenced by a biomedical paradigm focusing on the individual patient, and other paradigms with particular assumptions and beliefs, e.g. about causes and solutions to severe mental illness. How the work is organized will also influence culture, and vice versa.
  The interviews reveal that many NOK feel offended, ignored and treated as the cause of the patient’s illness. Many of these negative experiences seem avoidable. A change in attitude towards caregivers is needed in mental health care to value the caregivers’ role [1, 5, 7, 10, 15, 17]. If culture influences the help that the patient receives in a negative way, more active measures than guidelines need to be taken. Ethics reflection groups and ethics committees may strengthen awareness and knowledge on the role of NOK, on legal regulations and on ethical dilemmas related to confidentiality, and the role of NOK in clinical practice. This work may over time change culture more effectively than guidelines. Furthermore, active measures must be taken by the professionals, by administrators, policy makers and the ones responsible for education, together with patients and NOK.

## Conclusions

Although health care organisers and next of kin organisations for many years have emphasised a need to strengthen health care’s involvement of family members of seriously mentally ill persons, the involvement is still far from satisfactory. This fact is a threat to the health of the next of kin, to the patient and to an effective use of formal and informal health care. Our study shows that next of kin often are met in a way which adds to their anxiety, sorrow and guilt when their seriously ill family member is subjected to coercive measures. Active measures to change the mental health care culture, including increased knowledge in family ethics and law for health professionals, must be taken. Clinical ethics support may increase consciousness and competence among health care personnel and contribute with meeting-places where difficult ethical dilemmas concerning involvement of NOK can be discussed.

## Additional files

Additional file 1: Interview guide to next of kin of adult patients. (DOCX 23 kb)
Additional file 2: Interview guide to next of kin of adolescents. (DOCX 28 kb)

## Abbreviation

NOK: Next of kin

## Footnotes

1 Also described as informal caregivers, relatives, family or social network
