# Supplementary material for: Next of kin’s experiences of involvement during involuntary hospitalisation and coercion
Source: BMC Med Ethics. 2016 Nov 24;17:76. doi: 10.1186/s12910-016-0159-4 (PMC5121949; doi:10.1186/s12910-016-0159-4)
Supplement: Additional file 1: — Interview guide to next of kin of adult patients. (DOCX 23 kb) [file 12910_2016_159_MOESM1_ESM.docx]

## Interview guide for next of kin of adult patients

**EXPERIENCES WITH COERCION**

1. **What does coercion in mental health care mean to you?**
2. **What are your experiences with coercion in mental health care?**
3. **What is it like to be next of kin when coercion is used?**

- Did you and your relative experience the coercive situation the same way? (Have you talked about it?)

**4. Which (ethical?) challenges and dilemmas do you experience as next of kin in these kinds of situations?**

Possible follow-up questions

- Dilemmas related to the fact that the person does not see him/herself as sick, and does not want help.
- Confidentiality
- Child protective services
- Accessibility / discharge (too soon, not sufficiently planned?)

**VIEWS ON COERCION AND USE OF COERCION**

*Based on your experience, etc:*

**5. What is your view on the use of coercion in mental health care?**

*Possible follow-up questions*

Do you have examples of:

- Situations where you think coercion is the right choice?
- Situations where coercion is wrong, and where coercion should be used less or not used?

If so, what were the consequences? (ie. violation)

- Situations where there should be more coercion?
- How much persuasion and pressure is ok to use?
- Does the coercion have consequences on your relationship with your relative? How?
  Does the coercion have consequences on you relationship to the staff? How?

**6. Does the way the coercion is carried out matter?**

*For instance, is there a way to make it less of a violation and less of a burden on the recipient and on you? If so, how?*

**INVOLVEMENT AND PARTICIPATION OF NEXT OF KIN**

**7. To what degree and in which ways were/are you involved in the decision-making regarding coercion, before/during/after?**

Possible follow-up questions:

Based on their experiences:

- How was the dialogue/communication with the staff in the services you encountered?
- Did you get enough information?
- Were you able to influence the choices that were made, or that were important to you and perhaps also your relative, regarding coercion?
- Did you talk to the staff before the event (for instance the involuntary hospitalization), and were you taken into the decision-making process regarding coercion, talked to during the intervention, or offered a debriefing afterwards?
- Did the staff have enough knowledge about your rights, for instance confidentiality?
- Did confidentiality get in the way of your involvement?

**8. What should the role of next of kin be in situations where coercion is used?**

(Initiator, spokesperson, support, avoid responsibility)

**9. Do you think it is important that the closest next of kin be more involved in the coercive measures?**

(why/why not)

**HOW TO HANDLE DILEMMAS / CHALLENGES IN THE BEST WAY POSSIBLE**

Based on what we have talked about:

**10. What is the best way, or a better way, to handle next of kins’ dilemmas and challenges regarding coercion in the future?**

*Possible follow-up questions*

- At home or in the hospital
- For example the burden as a caretaker
- For example in situations where patients and next of kin disagree?
- For example when the patient does not want to allow involvement of next of kin

**11. How can next of kin be involved and included in a good way?**

*Possible follow-up questions:*

- Would debriefing before, during and after be helpful?
- Other suggestions for involvement?
- Could it be possible or of interest that patients and/or closest next of kin voluntarily participated in ethics reflection groups /clinical ethics committees in order to highlight the ethical dilemmas of the situation?

**Extra questions (measures towards reduced and better use of coercion):**

*Based on your experience:*

- Do you think it is possible to reduce and improve the use of coercion in mental health care?
- Based on your experience, in what way can the use of coercion be reduced and improved?

Possible follow-up questions:

- Would you - as next of kin - have liked to have more knowledge and skills regarding how to handle these kinds of situations better, for instance to avoid use of coercion. If so, what kinds of knowledge and skills?
- How does access to health care services affect the use of coercion?
- What help, support, and follow-up would you like from the health care services in these situations, in order to avoid use of coercion?
